# Use of Sodium-Glucose Transport Protein 2 (SGLT2) Inhibitor Remogliflozin and Possibility of Acute Kidney Injury in Type-2 Diabetes

**DOI:** 10.7759/cureus.32573

**Published:** 2022-12-15

**Authors:** Rajesh Jain, Natrajan Bhavatharini, Thangavel Saravanan, Veeraswamy Seshiah, Nishchal Jain

**Affiliations:** 1 Medicine, Jain Hospital and Research Center Pvt Ltd, Kanpur, IND; 2 Diabetology, SRC Diabetes Care Center, Erode, IND; 3 Nephrology, Abirami Kidney Care Private Limited, Erode, IND; 4 Diabetes and Endocrinology, The Tamil Nadu Dr. M.G.R. Medical University, Chennai, IND; 5 Medicine, Government Doon Medical College, Dehradun, IND

**Keywords:** acute renal failure and hemodialysis in icu, drug-induced acute renal failure, cardio-renal cascade, empagliflozin, sglt-2, sglt-2 inhibitor, diabetes type 2, acute renal injury, adverse event, remogliflozin

## Abstract

The major trials, e.g., EMPA-REG OUTCOME, CANVAS, and CREDENCE, showed the renal and cardiovascular benefit of sodium-glucose transport protein 2 (SGLT2) inhibitors. The SGLT2 inhibitors, Empagliflozin, Dapagliflozin, and Canagliflozin, have shown no significant adverse renal effects. Still, our patients with type 2 diabetes on Remogliflozin, a type of SGLT2 inhibitor approved in India for the treatment of diabetes, seems to cause acute tubular necrosis as confirmed by clinical and pathological evidence in our study. The two critical findings in our research include a consistent rise in hs-CRP and a pathologist's biopsy report, excluding other causes. Therefore, we need sizeable cardiovascular-renal outcome trials to ascertain the safety of Remogliflozin in future studies.

## Introduction

The sodium-glucose transport protein 2 (SGLT2) inhibitors or gliflozins are an excellent oral medication class that inhibits the SGLT2 transporter at the S1 segment of the proximal tubule, which is responsible for more than 90% of glucose reabsorption filtered through kidneys. Therefore, SGLT2i inhibits reabsorption and excretes glucose, resulting in lower blood glucose; it is also accountable for more diuresis and weight reduction in Type 2 diabetes patients. It reduces blood pressure and uric acid benefits beyond blood sugar control.

Till now, three drugs, Empagliflozin, Canagliflozin, and Dapagliflozin, have proved too significant in lowering cardiovascular endpoints and slowing the progression of chronic kidney disease (CKD) in type 2 diabetes through three landmark trials in diverse populations, e.g., EMPA-REG OUTCOME, CANVAS, and CREDENCE trials, Empagliflozin is the SGLT2 inhibitor, which reduces all-cause mortality highest and significantly among all SGLT2 inhibitors [1].

Many studies explored the use of SGLT2 inhibitors and the risk of acute kidney injury (AKI). A large study comparing the use of SGLT2 inhibitors and DPP inhibitors use and the risk of AKI and eGFR, this study shows the chance of AKI is lower (O.R.s,0.47) with the use of SGLT2 inhibitors [2]; in another study by Rampersad et al. [3] with 4,778 users, the use of SGLT2 inhibitors compared with 4,778 diverse oral hypoglycemic agents (OHA) consumers, no differences observed in the critical primary outcome (H.R., 0.64; 95% CI, 0.40-1.03; P = 0.06) using Intension to treat. In both, the group analyzed SGLT2 inhibitors were not associated with the increased adverse effect of risk for AKI.

A large meta-analysis trial by Neuen et al. and Menne et al. and a population cohort by Iskander et al. [4] showed that SGLT2 inhibitors reduced AKI risk in OPD set up patients. Hospitalized cases both cause modest diuresis and weight loss, lower blood pressure, and uric acid in patients with type 2 diabetes mellitus (T2DM), thus outspreading benefits outside glycemic control.

In most cases, the side effects of SGLT2 inhibitor use are more related to hypovolemia because of their activities due to diuresis, reduced AKI incidence with SGLT2 inhibitors in type 2 diabetes, and renoprotection might be attributed to the following reasons [5]: 1) release of NO-dependent vasodilation and reduction of AKI with increased perfusion, 2) release of vascular endothelial (GFA) growth factor and 3) reduction in renal fibrosis.

Therefore, we have considerable evidence that the SGLT2 inhibitor's clinical use in Type 2 diabetes does not significantly affect AKI. Still, instead, provide renal protection, and not only this, it offers long-term benefits by reducing the risk for dialysis, renal replacement therapy, and mortality in type 2 diabetes with CKD [6].

But at present, data on AKI related to SGLT2 inhibitor Remogliflozin are lacking. More study is needed to determine the adverse effects of Remogliflozin; outcome data with Remogliflozin is lacking in the literature, and reporting of renal dysfunction or AKI may be under-reported in the clinical setting. The actual mechanism of SGLT2 inhibitors leading to AKI is not known, as diuresis follows of inhibition of SGLT2 receptors in proximal tubules, ultimately leading to an increase in osmolarity and dehydration.

But we have glucose transporter GLUT9b located in proximal renal tubules, which reabsorbed glucose in exchange for uric acids. This results in uricosuria, which also decreases blood pressure. As a result, an increase in uric acid in the tubules may be implicated in AKI, and heat stress and dehydration contribute to it [7].

Another pathway for renal injury is that high glucose excretion in proximal tubules S1 segment leads to more glucose to the S3 element and SGLT1 receptors still are active for more glucose uptake, which may activate gene aldose reductase turn, leads to more production of fructose and sorbitol. The fructokinase is present here, which leads to more uric acid production and chemo kinins release, which further causes inflammation and oxidative stress leading to acute tubule injury [8].

The accumulation of sorbitol and fructose also causes a lower level of Myo-inositol in hyperglycemia, and that may lead to complications of diabetes, especially AKI [9]. Remogliflozin etabonate is approved in India by Drug Authority as an alternative treatment option for glycemic control in T2DM. Still, more extensive trials have not documented its safety and cardiovascular outcome on the cardiorenal system [10]. The other benefits of SGLT2 inhibitors on through a meta-analysis of eight studies for T2DM and NAFLD results to improve transaminase levels, fatty liver, and fibrosis, along with providing additional metabolic effects [11].

## Case presentation

A 57-year male known case of diabetes mellitus was received from outside the hospital with complaints of bilateral pedal edema, puffiness of the face, decreased urine output, burning micturition, and dysuria.

Differential diagnosis: type-2 diabetes mellitus with rapidly progressive renal failure with volume overload with metabolic acidosis and hyponatremia. The initiation of hemodialysis was done on November 26, 2020, the right internal jugular catheter started the same day, and a renal biopsy was done on November 27, 2020, which showed severe acute tubular necrosis. 

The patient presented with chief complaints of bilateral pedal edema, puffiness of the face, decreased urine output, and burning micturition. Vitals were normal; temperature 98.6º F, pulse rate 68/min, respiratory rate 20/min, blood pressure: 140/90 mmHg, and SpO_2_ 98% at rest. No history of hypotension or pedal edema or use of antibiotics/NSAIDs/ACEI/ARB/diuretic use in the past/present; no family history of diabetes or hypertension; urine MSU showed no bacterial Infection; no history of diarrhea. 

A patient with type 2 diabetes was well-controlled blood sugar for five years; presented to us with swelling of the lower limb after consuming Tab Remogliflozin 100 mg once a day for one month; the patient was earlier on a Tab of Pioglitazone 15 mg once a day for last many years, but he has changed to Tab Remogliflozin 100 mg as he gained around 8 kg of weight on Pioglitazone; therefore, he has been advised to stopped Pioglitazone and started with Remogliflozin 100 mg intending to reduce weight, the patient did not have in the past any episode of breathlessness or hypoglycemia or hypertension or dehydration. He is agriculture by profession and works very hard in the field.

**Figure 1:**
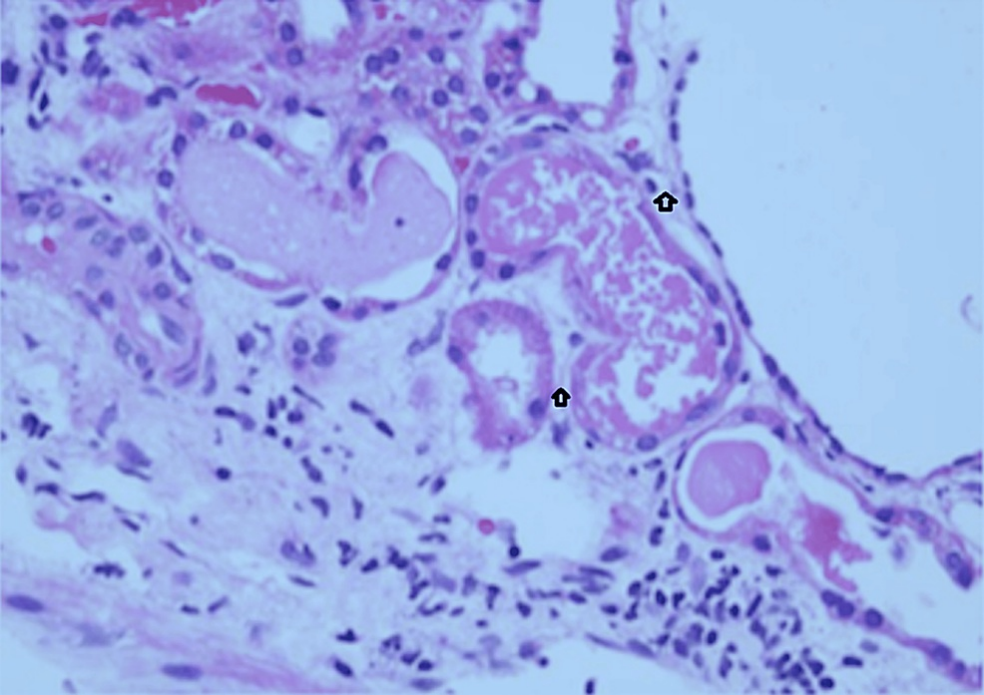
Acute tubular necrosis severe. Components of the core biopsy of renal cortex, acute tubular injury, severe; foci of acute tubular necrosis present; interstitium: expanded by edema and scattered lympomononuclear infiltrates. Overall interstitial fibrosis and tubular atrophy: focal (<5%). There are no diabetic nephropathy features on light microscopy. Image credit: Rajesh Jain

**Figure 2:**
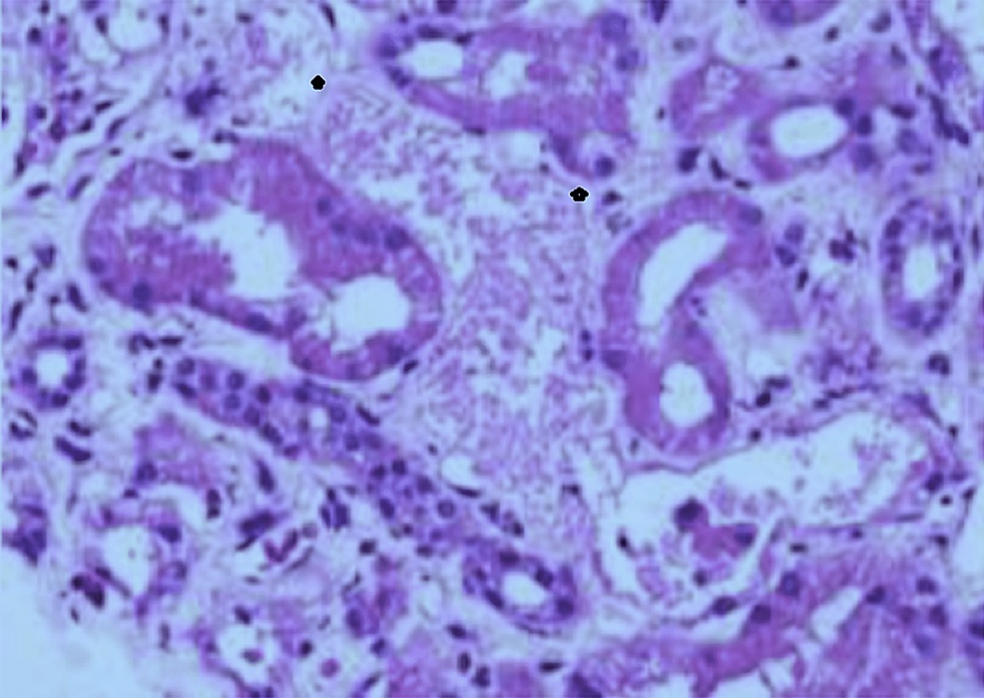
Acute tubular necrosis severe and persistent. Tubules: acute tubular injury, severe; foci of acute tubular necrosis present; interstitium: expanded by edema and scattered lympomononuclear infiltrates. Overall interstitial fibrosis and tubular atrophy: focal (<5%). Image credit: Rajesh Jain

Patient renal parameters, e.g., S creatinine increased from 1.4 mg/dL to 3.8 mg/dL and eGFR 18.2 mL/min/1.73 mEq/L within a week of Pedal edema, and consistent elevation of hs-CRP from 5.5 mg/L to 38 mg/L within one week of consultation; drug Remogliflozin was stopped after this episode was referred to a Kidney specialist, where he was admitted, and a biopsy was taken, and acute kidney necrosis was diagnosed without diabetes nephropathy (Figures 1, 2). The immunoglobulins IgA, IgG, IgM, Kappa, and Lambda light chains were within normal range; complement components C3c and C1q were also within normal range.

On evaluation, the patient had increased renal parameters; blood urea 49 mg/dL, serum creatinine 3.73 mg/dL (0.8-1.3 mg/dL), eGFR 18.2 mL/min/1.73 m^2 ^(90-120 mL/min/1.73 m^2^, hyperuricemia uric acid 6.78 mg/dL (3.5-7.2 mg/dL), hyponatremia sodium 125.0 mEq/L (135-145 mEq/L), metabolic acidosis HCO_3_ 17.9 mEq/L (21-28 mEq/L, lymphopenia lymphocyte 1.13 (1.0-4.8 per milliliter), hypoalbuminemia, albumin 2.36 g/dL (3.5-5.5 g/dL), microhematuria and proteinuria+++ with no bacteriuria.

Management in the hospital

Ultrasound abdomen shows a normal-sized kidney, bulky kidney. he was diagnosed with rapidly progressive renal failure. After the renal biopsy was sent for examination, the patient's treatment started on an intravenous antibiotic, Cefoperazone with Sulbactam, intravenous diuretic, and hyperuricemia was managed with febuxostat 40 mg two times a day, hyponatremia was organized with 3% NaCl 100 mL intravenous two times a day, metabolic acidosis was managed with sodium bicarbonate 1mmol/kg (1mL/kg 8.4% solution) followed by 0.5mmol/kg (0.5mL/kg 8.4% solution) given at 10-minute intervals; Tolvaptan 30 mg once a day for two days. HRCT chest was done on November 26 and reported bilateral mild pleural effusion. The patient initiated hemodialysis on November 26, 2020 through the suitable internal jugular catheter. In the view of the patient with a normal-sized kidney with a rapid decline of GFR, renal tissue was sent for renal biopsy; intra and post-procedure periods were uneventful. The serial CRP level was elevated during the hospital stay, and the patient was managed with a higher antibiotic (Meropenem). The patient was put on insulin to control blood sugar and discharged after relief.

The patient has again admitted with the same problems after two months; right-sided permanent tunneled catheter insertion was done again on February 15, 2021. Advised renal replacement therapy in the form of hemodialysis till renal function improved. The Patient had an elevated PTH level and was diagnosed with secondary hyperparathyroidism managed with calcitriol. The patient had six sessions of hemodialysis. The patient's urine output improved. The patient was symptomatically better and discharged in stable condition. The patient was admitted twice because of renal dysfunction, and a biopsy was repeated on February 15, 2021. On that basis, he was diagnosed with acute tubular necrosis with a severely persistent type; he was discharged after hemodialysis.

The patient's renal parameter improved serum creatinine 3.05 mg/dL. The patient was symptomatically better and discharged in stable condition. Recently the patient's follow up was done, and his serum creatinine varies in the range is 1.1-1.49 mg/dL, eGFR (78-64) mL/min/1.73m^2^; his problem increased when he switched to a non-vegetarian diet.

Biopsy report on admission shows severe acute tubular necrosis. Components of the core biopsy of renal cortex, acute tubular injury, severe; foci of acute tubular necrosis present; interstitium: expanded by edema and scattered lympomononuclear infiltrates. Overall interstitial fibrosis and tubular atrophy: focal (<5%). There are no diabetic nephropathy features on light microscopy.

Biopsy report after two months: acute tubular necrosis is severe and persistent. Tubules: acute tubular injury, severe; foci of acute tubular necrosis present, interstitial: expanded by edema and scattered lympomononuclear infiltrates, overall interstitial fibrosis and tubular atrophy: focal (<5%). Glomerular compartment: total number of glomeruli 5, no globally sclerosed, no segmentally sclerosed, non-sclerosed normocellular with regular capillary loops.

## Discussion

The renal adverse effects of Remogliflozin are not documented in the literature. Therefore, this case study provides further investigations for AKI as confirmed by biopsy. Reports of such cases submitted to the USFDA may be the tip of the iceberg, and we believe many such cases remain underreported in low-resource countries like India. Although such cases led the FDA to issue a warning for monitoring renal function within an hour of the start of therapy and later on, the notice was withdrawn because of the outcome trial of EMPA-REG OUTCOME, CANVAS, and CREDENCE, which showed the renal cardiovascular and renal benefit of these SGLT2 inhibitors but remogliflozin is a new drug recently approved in India for type 2 diabetes treatment.

SGLT2 Inhibitors like Empagliflozin, Canagliflozin, and Dapagliflozin have been studied widely for cardiovascular and renal outcomes and found to be protective. Although the SGLT2 Inhibitor Remogliflozin has shown blood-lowering effects in type 2 diabetes, cardiovascular and renal outcomes trials are still not done with this drug. Therefore, the researchers have doubt it may lead to renal adverse effects as in this patient; two critical findings for AKI, in this case, is a consistent increase in the hs-CRP and a biopsy of renal tissue; we hope that this case study can alarm physician treating type 2 diabetes to report such cases and extensive cardiovascular outcome study should be undertaken to prove its safety or benefits further as done with other classes of SGLT2 inhibitors.

## Conclusions

In this case, two critical findings were one rapid CRP increase and a biopsy consistent with an AKI. Remoglifloazin might be associated with this AKI in this case. Therefore, more study is needed to prove the safety of this SGLT 2 inhibitor in renal dysfunction or risk for CKD. The Indian federal drug authority approved Remogliflozin in 2019 for use in type 2 diabetes, but the drug is still not approved in Europe and USA. Therefore, we need a large CV renal outcomes trial in diabetes with CKD patients to ascertain the safety and benefits of Remogliflozin.
